# Supplementary material for: A Machine-Learning Tool Concurrently Models Single Omics and Phenome Data for Functional Subtyping and Personalized Cancer Medicine
Source: Cancers (Basel). 2020 Sep 30;12(10):2811. doi: 10.3390/cancers12102811 (PMC7601761; doi:10.3390/cancers12102811)
Supplement: Supplementary file 1 [file cancers-12-02811-s001.zip › Supplementary files/20200928_20200912_20200918_20200726_20190930-20190712-Functional-Subtyping-SupplementaryFile_final.docx]

**Supplementary Information**

A Machine-Learning Tool Concurrently Models Single Omics and Phenome Data for Functional Subtyping and Personalized Cancer Medicine

Gift Nyamundanda ^1^, Katherine Eason ^1^, Justin Guinney ^2^, Christopher J. Lord ^3^ and
Anguraj Sadanandam ^1,^*

^1^ Division of Molecular Pathology, The Institute of Cancer Research, London, SW3 6JB, UK; gift.nyamundanda@icr.ac.uk (G.N.); kate.eason@icr.ac.uk (K.E.)

^2^ Sage Bionetworks, Seattle, WA 98121, USA; justin.guinney@sagebase.org

^3.^ The Breast Cancer Now Toby Robins Research Centre, The Institute of Cancer Research, London,

SW3 6JB, UK; Chris.lord@icr.ac.uk

***** Correspondence: [Anguraj.sadanandam@icr.ac.uk](mailto:Anguraj.sadanandam@icr.ac.uk); Tel.: +44-2034376440

**SUPPLEMENTARY NOTES**

**Permutation based assessment of UF-subtypes robustness**

The class labels for UF-subtypes were randomly shuffled (permuted) a thousand times and the Euclidean distance of each sample to its corresponding subtype mean (centroid) was computed for each permutation. The observed distances of the true subtypes (distances without shuffling) were compared to the distribution of the distances of the permuted subtypes (**Figure S2B)**. The observed distances for each subtype are expected to be small compared to the distances of random permutations. Here, we set 1% significance level as a cut-off point to identify subtypes whose distances to the centroid is close to random. **Figure S2B** shows that due to few samples UFs-6 is not a very robust UF-subtype; hence, we did not consider this subtype in further analysis.

**Development of classifiers**

In order to assign new samples into UF-subtypes, we developed classifiers based on prediction analysis of microarray (PAM [1]) centroids for both 576 genes (**Table S3**) selected by *PhenMap*. PAM centroid represent scaled average expression of the signature in each subtype [6]. Using-five fold cross validation; PAM centroid with the least misclassification error rate (retaining all the genes selected by *PhenMap*) was generated for both classifiers. The expression pattern of a new sample was correlated to the PAM centroid and assigned to the subtype with highest Pearson correlation coefficients.

**Clustering cell-lines into three drug response groups**

In an effort to classify cell-lines into three drug response groups; high (H), moderate (M) and low (L) sensitivity, the drug response information (-log GI_50_) of the 37 BC cell-lines were clustered separately (using K-means) for each drug. The three response groups are shown in **Figures 1E** - **F and Figure S3C**.

**Runtime profiles for *PhenMap***

Runtime profiles were generated by fitting the current version of *PhenMap* (v1.0) to different simulated datasets using iMac (Apple Inc.) with Macintosh OS X Yosemite (version 10.10.5) and one central processing unit with four cores each of 4 gigabits of random access memory. The following simulation study was carried out by varying the sample size (*n*) and the number of features (*p*). Firstly, we simulated *n* set of samples randomly assigned into two groups using binomial distribution with probability of 0.5. Secondly, we simulated expression data from a multivariate Gaussian distribution with the two randomly assigned sample groups having different mean and covariance. Finally, a continuous covariate is further simulated from a standard normal distribution. This covariate was combined with the binary variable (generated above), associated with the two randomly assigned sample groups, to define two covariates matched to the *n* samples.

**Figure S6A** shows that the runtime profiles of running *PhenMap* (v1.0) on simulated dataset of 20-50 samples, 2 covariates and 50-1000 features increases exponentially with the number of features, starting from 3 minutes to 400 minutes. Nevertheless, when we adopted a spike and slab prior [2] (a mixture of very peaky and broad Gaussians) instead of the automatic relevance determination (ARD) sparsity-inducing prior over the loadings matrix the new version of the *PhenMap* (v2.0) has improved the runtime to less than two minutes for at most 50 samples and 1000 features (**Figure S6B**).

**SUPPLEMENTARY METHODS**

**The likelihood of the model for *PhenMap***

The probability of observing both the data, **Y**, and the CMVs, **U**, given the parameters, **Θ** = ( **W**, **Σ**, **β**, **Φ** ), i.e., the full augmented data likelihood under *PhenMap*, is

*p*( **Y**, **U** | **Θ** ) = $\prod_{i=1}^{n}$ ( MVN*_p_*[ *y*_i_ | **W***u*_i_, **Σ** ] $\star$ MVN*_q_*[ *u*_i_ | **β***x*_i_, **Φ** ] ). (1)

Bayesian methodology was adopted to estimate this model as it is a flexible approach to encourage sparsity in some of the parameters in **Θ** and also provides all information about the distribution of **Θ**.

**Identifiability of the model in *PhenMap***

The most important limitation associated with the posterior distribution of the model in *PhenMap* is the identifiability issue [3], i.e., orthogonal rotation of the loadings and CMVs of *PhenMap* can result in the same data distribution. To solve this, a post-processing approach is taken in which the estimated loadings matrix is rotated to match the loadings estimated at the start of the chain after burn-in period.

**Colorectal cancer subtypes**

Colorectal cancer (CRCAssigner) subtypes were calculated using PAM centroids from Sadanandam et al., publication [4].

**Other statistical tests**

Kruskal Wallis test was used to test for association between subtypes and drug response information. Fisher’s exact test was used to assess association between categorical variables whilst enrichment analysis was done using hyper-geometric test. Prognosis analysis was performed using log-rank test. To assess the overall effect of each CMV in multivariate Cox regression, Analysis of variance (ANOVA) was performed. Significance level was set at 5% unless stated. Disease specific survival for METABRIC data [10] was calculated using overall survival, with death from disease treated as event whilst censoring other causes of death. The survival information for METABRIC data was from cBioPortal (<http://www.cbioportal.org/datasets>; access date: 2017/05/10). Kaplan-Meier curve and log rank test were performed.

**SUPPLEMENTARY FIGURE LEGENDS**

**Figure S1. Assessment of convergence and fit of *PhenMap* model on the training data (gene expression of BC cell lines)**. (**A**) Trace plots for regression coefficients (β), (**B**) CMVs (U) of randomly selected samples, (**C**) variance estimates (σ^2^), and (**D**) loadings (W) of randomly selected genes. (**E**) The plot of the mean absolute deviations (MAD) values between the covariance of the data generated from the posterior predictive distribution and the covariance of the observed (training) data. The vertical dashed red line denotes the cut-off set at one for MAD.

**Figure S2. Robustness of the six UF-subtypes and comparison to non-negative matrix factorization (NMF).** (**A**) Cophenetic coefficients and silhouette width plot for different number of UF-subtypes from 37 breast cancer cell lines. High cophenetic coefficients or silhouette width indicate better clustering. (**B**) Permutation plot showing the average distances of samples to their centroids after random permutations (in grey) of the subtypes and the average distance of the UF-subtypes. (**C**) Cophenetic coefficients and silhouette width plot for different number of NMF subtypes from 37 breast cancer cell lines. High cophenetic coefficients or silhouette width indicate better clustering. (**D**) A plot showing the CMV-1 and 2 (the grey dashed lines represent the origin or zero of both CMVs) and NMF subtypes. Six different colors identify the six UF-subtypes, whereas three NMF-subtypes (NMF-1, -2 and -3) are denoted by dashed blue circles.

**Figure S3. The UF-subtypes are associated with the known intrinsic BC, colorectal cancer subtypes, and drug response. (A** - **B**) Heatmap of p-values from hyper-geometric test enrichment analysis between the UF-subtypes and (**A**) the intrinsic BC subtypes [5] and (**B**) the colorectal cancer subtypes (CRCAssigner) [4]. Red colour identifies significant association. TA: transit-amplifying, SL: stem-like, INF: inflammatory, GL: goblet-like, and En: enterocyte. (**C)** A plot that compares the scaled -log_10_ [GI_50_] values for etoposide and fascaplysin in the 37 training BC cell lines highlighting four known intrinsic breast cancer subtypes. The grey dashed lines *H, M* and *L* represents high, moderate and low sensitivity of the cell lines to the drugs, respectively. (**D**) Heatmap showing the expression pattern of top genes specific to each UF-subtype. The top genes are those with the biggest difference between the largest and the second largest centroids across subtypes.

**Figure S4. Prognosis of predicted UF-subtypes and context specific functional subtyping of the training data.** (**A**) Disease specific survival (DSS) of the five UF-subtypes predicted in 1904 METABRIC BC samples [6]. (**B**) Cophenetic coefficients and silhouette width plot for different number of CMV-1 functional subtypes in the training data. High cophenetic coefficients or silhouette width indicate better clustering. (**C**) Heatmap of p-values from hyper-geometric test enrichment analysis between the UF-subtypes and the six CMV-1 functional subtypes. Red colour identifies significant association. (**D**) Heatmap showing the expression pattern of genes specific to the two CMV-2 functional subtypes across the scores of the second CMV, the six UF-subtypes and five intrinsic subtypes. The top genes are those with largest difference between the largest and the second largest centroids across subtypes. (**E**) Proportional plot comparing the proportions of the two CMV-2 functional subtypes in BC cell lines and patient tumors from GSE42568 [7] dataset.

**Figure S5. Results of applying *PhenMap* to BC patients samples.** (**A** – **B**) BIC plots to identify the optimal number of CMVs and general subtypes in gene expression of 101 breast tumors from GSE42568 [7] dataset, respectively. The red dashed lines identify the optimal number of CMVs or general (G)-subtypes. (**C**) Heatmap showing association of the G-subtypes with ER, grade and age.

**Figure S6: Runtime profiles for *PhenMap* and its modified version.** Run time profiles for (**A**) *PhenMap (v1.0)* and (**B**) *PhenMap (v2.0)* applied to several simulated expression data sets varying sample size (*n*) and number of features (*p*).

**SUPPLEMENTARY TABLES**

**All supplementary tables are available separately as Excel Worksheets.**

**Table S1**. **Datasets and drug response data.** (**A)** A list of all datasets used in this manuscript. The table includes the platforms, sample types, normalization methods, number of samples, sample sources and publications. **(B)** Drug response information (-log10 [GI50] values) for the 37 breast cancer cell lines used as training data. The table includes CMV scores, UF-subtypes and intrinsic subtypes.

**Table S2**. **Association of UF-subtypes with drug response.** Association of UF-subtypes with (**A**) etoposide and (**B**) fascaplysin response groups. The response groups were identified by clustering -log10 [GI50] values for etoposide and fascaplysin into three-response groups separately.

**Table S3. UF-subtype gene signature.** The 576-gene signature PAM centroids for the five UF-subtypes.

**Table S4. Subtype information.** Predicted (**A**) UF-subtypes and CMV2-subtypes for GSE42568 and (**B**) UF-subtypes for METABRIC data.

**Table S5. PAM centroids**. PAM centroids for the two context-specific metavariable (CMV)-2 subtypes.

**Table S6. Clinical information and CMVs for GSE42568 *PhenMap* results.**

## REFERENCES

1. Tibshirani R, Hastie T, Narasimhan B, Chu G. Diagnosis of multiple cancer types by shrunken centroids of gene expression. Proc Natl Acad Sci. 2002;99:6567–72.

2. Ishwaran H, Rao JS. Spike and slab variable selection: Frequentist and bayesian strategies. Ann Stat. 2005;33:730–73.

3. Shapiro A. Identifiability of factor analysis: some results and open problems. Linear Algebra Appl. 1985;70:1–7.

4. Sadanandam a, Lyssiotis C a, Homicsko K, Collisson E a, Gibb WJ, Wullschleger S, et al. A colorectal cancer classification system that associates cellular phenotype and responses to therapy. Nat Med. 2013;19:619–25.

5. Heiser LM, Sadanandam A, Kuo W-L, Benz SC, Goldstein TC, Ng S, et al. Subtype and pathway specific responses to anticancer compounds in breast cancer. Proc Natl Acad Sci. 2012;109:2724–9.

6. Curtis C, Shah SP, Chin SF, Turashvili G, Rueda OM, Dunning MJ, et al. The genomic and transcriptomic architecture of 2,000 breast tumours reveals novel subgroups. Nature. 2012;486:346–52.

7. Clarke C, Madden SF, Doolan P, Aherne ST, Joyce H, O’Driscoll L, et al. Correlating

transcriptional networks to breast cancer survival: A large-scale coexpression analysis.

Carcinogenesis. 2013;34:2300–8.
